# A Case of Organic Disease of the Stomach, with the Appearances after Death

**Published:** 1829

**Authors:** Samuel Martin

**Affiliations:** Col. Reg. Chir. Lond. Soc. of Morgan County, Ohio


*November*, 1829.

---

Art. IV.—*A Case of Organic disease of the Stomach, with the appearances after death.* By Samuel Martin, M. D. Col. Reg. Chir. Lond. Soc. of Morgan County, Ohio.

Samuel McCune, I suppose about 60 years old, and till within some months a strong and hard working man, mentioned to me in October 1828, that he had had, for a considerable length of time (perhaps nearly a year) a kind of burning pain, not however easily described, at the stomach; this pain was sure to come on soon after eating, but also came on at other times; after one or two eructations it was always relieved for a while. The tongue was clean, the appetite good, no sickness at stomach, and the bowels as he said, regular.—He said he felt "a sort of squeezing as if something was trying to pass through the stomach and could not;" and that he was "getting weaker and weaker."

I told him he might try for a while, a mixture of laudanum, sulphuric æther and tincture of asafœtida, which he did; I afterwards only saw him by chance, but I heard that the medicine had no good effect, indeed very little effect whatever, tho' taken in considerable doses; and that several other medcines he had tried, had very little effect of any kind: from these circumstances I began to suspect an alteration of structure, perhaps scirrhus, and mentioned it to the family, which was the cause of the patients desiring to be examined after death. Afterwards he tried by my desire extract of conium, for a short time, without effect; he used also for a short time the runnet of a calf with a little alleviation of his sufferings.

I saw him about three weeks before death; at this time he was greatly emaciated; the stomach could be felt, small and hard, and put into motion by the pulsations of the descending aorta; the abdomen in that part was a little protruded. Soon after this he vomited almost every thing that he ate; and sometimes, as I was told, a dark matter resembling coffee grounds. He became continually more and more emaciated till he died. His death, which had daily been looked for, thro' the last fortnight, happened on the 18th of July of this year.

A few hours after death, the stomach was examined; an incision was made through the abdominal parieties from the ensiform cartilage to the spine of the left ilium; the angular flap thus made being turned over the side, the stomach was exposed to view; it had a firm, white, shining appearance, like fascia; and was quite hard to the touch. Being removed from the body and placed on a table, it was laid open, by an incision along the line of the smaller curvature, from the rephagus to the pylorus. A small quantity of brownish fluid escaped.

About one eighth part only of the whole organ appeared exempt from disease, and capable of distention: this was on the smaller curvature and near the osophagus. I do not think the stomach, even by a forcible injection, could have been made to hold half a pint.

The stomach being spread open, the remaining seven eighths of its interior appeared studded over with soft hillocks of various and irregular shapes, some as large as a hazlenut and others of 3 or 4 times that size; resembling in colour the cineritious part of the brain, perhaps inclining more to a brown; these hillocks, as I have called them, were so close together, that when the stomach was closed up, as in its state before being cut into, their sides touched and fitted together, and then the internal surface of the stomach, viewed through the incised opening, appeared only a little uneven.

On cutting into these lumps down to their bases, it appeared that the softness and the brownish colour reached only to a small depth, the color then changing gradually to that of cartilage, and the substance growing gradually harder, till arriving near the external surface of the stomach, they were of a cartilaginous and almost tendinous hardness, and could scarcely be indented by the handle or even the point of the scalpel.

It was an inch, I should say, from the top to the base of some of the larger lumps, or in other words, the wall of the stomach was there an inch thick. I observed no radiated but in the hardest part a lamellated appearance. It was at the pyloric extremity that the greatest hardness and near it the greatest thickness was. I think from some spots looking a little inflamed on the inner surface of the stomach, that if the patient could have lived a little longer, ulceration would have commenced.

The mesenteric glands in the vicinity partook of the disease. The liver was of a natural color and consistence; but there were two small cysts on its upper surface, which were not opened; it being understood that no parts except the stomach were to be meddled with; and I suppose they were of a common kind and had nothing to do with the patients sufferings. This is the only opportunity afforded me for 12 years of examining the human viscera; but I thing the spleen was less firm than natural, as it was ruptured whilst drawing it forward very gently with my fingers; and that the small intestines were rather firmer and thicker than natural.

I would remark, that I should suppose such a formation as here described would take place only in a contractile bag, like the stomach, possessed of several distinct tunics; and that it may be accounted for thus; For some time after the vessels had taken on the diseased action, the stomach remained capable of contraction and distention. The contraction of the muscular coat would throw the mucous into wrinkles, the crevices were the clefts between the lumps I have described, and the cartilaginous matter was deposited by the vessels in the raised parts of the wrinkled surface,—between the brown substance, which was the diseased and thickened mucous membrane, and the fascia like external surface, which was the diseased peritoneal coat—in the mean time the muscular fibre disappeared gradually.

